# Selected Serum Markers Associated with Pathogenesis and Clinical Course of Type 1 Diabetes in Pediatric Patients—The Effect of Disease Duration

**DOI:** 10.3390/jcm12062151

**Published:** 2023-03-09

**Authors:** Agnieszka Ochocińska, Marta Wysocka-Mincewicz, Jolanta Świderska, Bożena Cukrowska

**Affiliations:** 1 Department of Biochemistry, Radioimmunology and Experimental Medicine, The Children’s Memorial Health Institute, Aleja Dzieci Polskich 20, 04-730 Warsaw, Poland; 2 Clinic of Endocrinology and Diabetology, The Children’s Memorial Health Institute, Aleja Dzieci Polskich 20, 04-730 Warsaw, Poland; 3 Department of Pathomorphology, The Children’s Memorial Health Institute, Aleja Dzieci Polskich 20, 04-730 Warsaw, Poland

**Keywords:** markers, type 1 diabetes, disease duration, metabolic memory

## Abstract

Biochemical abnormalities in the course of type 1 diabetes (T1D) may cause the production/activation of various proteins and peptides influencing treatment and causing a risk of complications. The aim of this study was to assess concentrations of selected serum substances involved in the pathogenesis and course of T1D and to correlate their concentrations with the duration of T1D. The study included patients with T1D (*n* = 156) at the age of 3–17, who were divided according to the duration of the disease into those newly diagnosed (*n* = 30), diagnosed after 3–5 (*n* = 77), 6–7 (*n* = 25), and over 7 (*n* = 24) years from the onset of T1D, and age-matched healthy controls (*n* = 30). Concentrations of amylin (IAPP), proamylin (proIAPP), catestatin (CST), chromogranin A (ChgA), nerve growth factor (NFG), platelet-activating factor (PAF), uromodulin (UMOD), and intestinal fatty acid binding protein (I-FABP) were measured in sera using immunoenzymatic tests. There were significant differences in concentrations of all the substances except UMOD and NGF between T1D patients and healthy children. The duration of the disease affected concentrations of CST, ChgA, PAF, and NGF, i.e., proteins/peptides which could have an impact on the course of T1D and the development of complications. In long-term patients, a decrease in concentrations of CST and ChgA, and an increase in PAF concentrations were found. In the case of NGF, a decrease was observed after the initial high values, followed by an increase over 7 years after T1D diagnosis. Concluding, the results show that concentrations of selected serum indicators may change in the course of T1D. Further studies are needed to establish whether these indicators could be used in the context of predicting long-term complications.

## 1. Introduction

Type 1 diabetes mellitus (T1D)—one of the most widespread diseases of our time—is a chronic disease that results from the autoimmune destruction of insulin-producing β-cells in the islets of Langerhans in the pancreas [1]. By 2022 there were 8.75 million individuals worldwide with T1D (119,995 in Poland), 1.52 million (15,220 in Poland) were younger than 20 years of age [2]. Looking at worldwide data, 530,000 new cases of T1D were diagnosed across all ages, of which 201,000 were under the age of 20. Therefore, the scale of the problem is very large [2]. The discovery and development of more physiologically active insulins applied together with continuous subcutaneous infusion pumps, and the improvement of T1D care contributed to a significant extension of T1D patients’ life span and quality. However, untreated or improperly treated disease causes many complications, both acute and chronic [3,4]. Pathogenesis of T1D has not been fully elucidated so far, and factors inducting the disease are still the subjects of many studies [5]. It is already known that great importance in the pathogenesis of diabetic complications is not only the current metabolic control but also the careful and intensive treatment of the disease from the moment of its diagnosis [6].

It is proven that changes in metabolic control, in any of the stages of the diabetes course, influence complication risks in the future, even after 20–30 years of the duration of the disease. This phenomenon, called “metabolic memory”, is probably the consequence of an increase in oxidative stress factors caused by hyperglycemia, is partly irreversible, and persists even after normalization of glycemia [7].

Abnormal glucose concentrations and metabolites generated as a result of excess hormones activate many unfavorable metabolic processes (non-enzymatic glycation of proteins, changes of the polyol and hexosamine pathways, activation of protein kinase C, oxidative stress, and tissue hypoxia). These pathological pathways lead to the failure of various organs, especially the eyes, kidneys, nerves, heart, and blood vessels. Disturbances in growth (short height or overgrowth) and puberty (premature or delayed puberty) can also be other types of T1D complications in children [8]. Late complications in the first 5 years of diabetes in both pediatric and adult patients are sporadic, but their number increases with the duration of the disease. Macrovascular changes usually manifest themselves clinically after the onset of microangiopathic changes, especially in diabetic nephropathy. However, before the clinical manifestation of structural changes in the vessels, functional changes in the microcirculation occur first. These changes are reversible, and therefore early markers are being sought to identify the early stages of biochemical disorders preceding endothelial dysfunction [6,8].

There are still no tools for diagnosing these early stages of the development of late complications, but only for the stages of functional changes preceding the appearance of permanent structural changes visible in imaging tests. Despite the availability of high-performance omics technologies, data inconsistency and a lack of unambiguous, highly promising protein markers are observed [9,10]. Postulated glycemic control refers to keeping blood glucose levels as close to the normal range as possible in order to prevent acute and chronic complications that would result from living with glucose levels significantly above or below the desired range. In practice, it can be achieved by measuring fasting blood glucose or using glycated hemoglobin (HbA1c), a measurement of average blood glucose, over about three months. Currently, HbA1c has also been presented to play the role of an indicator of disease progression in diabetes. HbA1c has been shown to be directly related to the survival of patients with an approximately 30-year history of T1D [11]. However, it is known that HbA1c is not an ideal indicator of metabolic control [12,13,14]. 

Therefore, the aim of our study was to assess concentrations of selected active substances such as proteins, peptides, hormones, and others, the participation of which in the pathogenesis and course of diabetes and its complications is known or postulated, and to correlate concentrations of these indicators to the duration of T1D, as well as to compare these concentrations to those of a healthy population. We analyzed the levels of islet amyloid polypeptide/amylin (IAPP) and its prohormone—proamylin (proIAPP)—substances influencing the mechanisms of carbohydrate metabolism [15,16,17,18]; chromogranin A (ChA)—a supposed autoantigen in T1D [19,20,21]; and the product of its proteolysis catestatin (CST) [22,23], intestinal fatty acid binding protein (I-FABP)—a protein responsible for increased permeability of intestinal epithelial cells, showed to be increased in children with T1D [24]. We also included in our analysis substances potentially evaluable as indicators of late complications, neurological—nerve growth factor (NGF) [25], and vascular—platelet-activating factor (PAF) [26,27]. Finally, we assessed uromodulin, a protein secreted by the cells of the distal nephron tubules, strongly correlated with eGFR, which could be related to diabetic nephropathy in T1D patients [28]. 

## 2. Materials and Methods

### 2.1. Patients and Study Design

The study included 156 patients with T1D whose median age was 11 years of age (range: 3–17); 60 (46.6%) boys and 66 (52.4%) girls that were hospitalized at the Clinic of Endocrinology and Diabetology of the Children’s Memorial Health Institute in Warsaw. T1D was diagnosed according to the recommendations of the International Society for Pediatric and Adolescent Diabetes [29,30]. Routine serological tests were performed in The Central Laboratory (glucose, HbA1c) and in The Department of Biochemistry, Radioimmunology and Experimental Medicine (peptide-C, anti-glutamic decarboxylase (anti-GAD), anti-tyrosine phosphatase (anti-IA2), and anti-islet cell (ICA) antibodies) at the Children’s Memorial Health Institute. At least 2 autoantibodies (anti-GAD, anti-IA2, or ICA) were positive in T1D patients. T1D patients with current inflammation, hypoxia, and coexisting diseases were excluded from the study. A detailed biochemical status of the T1D patients is presented in Table 1. We have not observed the relationship of presented biochemical parameters with selected active substances assessed in this study (the analysis of the value of all r indicators in Spearman’s correlation analysis was <0.2). 

The T1D group consisted of 30 patients (17 girls and 13 boys) with newly diagnosed T1D (duration of diabetes < 3 months, at least one week after correcting the acid-base imbalance, i.e., normalization of blood gas results), and 126 patients (66 girls and 60 boys) with T1D lasting more than 3 years (median duration—5 years, range: 3–14). The group of long-term patients was divided according to how long it had been since onset in the following way: patients with no expected complications (first 3–5 years of disease; *n* = 77; 15 girls and 62 boys), patients with expected first biochemical changes indicating complications (6–7 years of disease, *n* = 25; 13 girls and 12 boys), and patients in which complications are highly probable (patients > 7 years of disease, *n* = 24; 13 girls and 11 boys). All of the patients included in the study had no documented neuropathy, hypertension, or retinopathy on the day of performing the biochemical analysis.

The control group consisted of 30 apparently healthy children (14 girls and 16 boys) at a median age of 8 years (range: 4–17 years) with no history of diabetes diagnosis and with the same exclusion criteria as the study group (no comorbidities of other causes, no signs of present inflammation, no clinically significant anemia, and no signs of hypoxia).

### 2.2. Methods

All active substances were assessed using the enzyme immunoassay ELISA (IAPP, proIAPP, NGF, ChgA, PAF—Cloud Clone Corp, Katy, USA; CTS—RayBio, Norcross, USA; UMOD—BioVendor, Brno, Czech Republic; I-FABP—Hycult Biotech Inc., Wayne, PA, USA) according to the test manufacturer’s instructions. The remaining biochemical tests, including HbA1c, were assessed by routine laboratory methods (Abbott Alinity ci-series assays).

### 2.3. Statistical Analysis

The minimum sample size was estimated at 86 participants using Epi Info 7 (available at: https://www.cdc.gov/epiinfo/index.html; accessed on 1 February 2023). Data were analyzed using Statistica v.10.0 software (StatSoft, Inc., Tulsa, OK, USA). Standard deviations of means were used as descriptive statistics. Normal distribution was checked using the Shapiro–Wilk test and revealed a non-normal distribution of data. Differences between two independent groups were tested by the Mann–Whitney U test and between three or more subgroups by Kruskal–Wallis ANOVA by Ranks for independent groups. Correlation analysis was done with the use of the Spearman rank correlation test. If the differences were significant, post hoc analysis using the Dunn–Bonferroni test was then performed. In all tests, *p*-values < 0.05 were considered significant. 

### 2.4. Ethical Approval

The study was approved by the Local Ethics Committee from the Children’s Memorial Health Institute (18/KBE/2019, date of approval: 24 April 2019) with the written informed consent obtained from participants over 16 years of age and/or their legal representative, as appropriate.

## 3. Results

### 3.1. Patient Characteristics

The subgroups distinguished on the basis of the duration of the disease statistically differed significantly (*p* < 0.05) in terms of biochemical parameters reflecting carbohydrate metabolism (glucose, HbA1C, insulin), lipid metabolism (HDL cholesterol and triglycerides), as well as reflecting kidney function (creatinine).

In the case of blood glucose and HbA1C, the highest concentrations were observed in newly diagnosed patients. With the duration of the disease, as a result of the implemented treatment, their concentrations decreased. However, after 7 years after the T1D diagnosis, an increase in the concentrations of both parameters was observed. Statistically significant differences between individual groups are listed in Table 1. Despite statistically significant differences between insulin concentrations in individual subgroups, all values were within the reference range of 4–16 mIU/L. 

Lipid profiles, regardless of the T1D duration, were within the target values recommended in the current recommendations [29]. In the case of HDL cholesterol and triglycerides, the values changed among T1D subgroups reaching a statistically significant difference, but without clinical significance.

Similarly, in the case of creatinine, statistically significant changes between the subgroups were not clinically significant.

### 3.2. Concentrations of the Selected Active Substances in T1D Patients and Healthy Controls 

**Figure 1:**
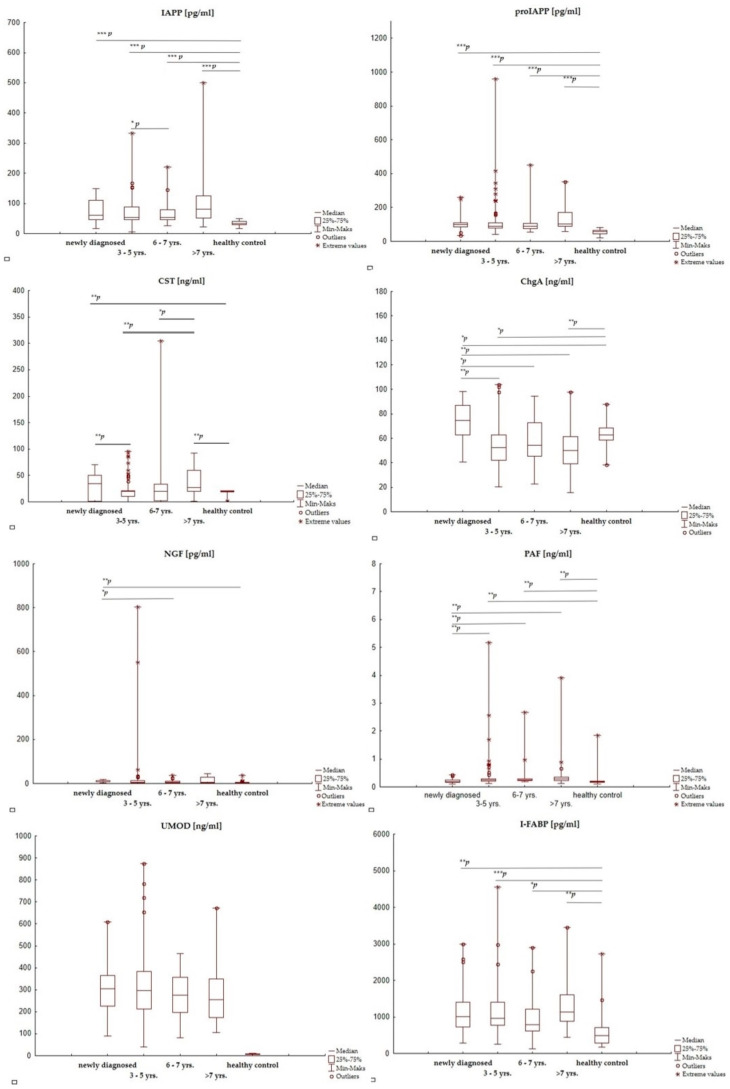
Selected active substances in T1D patients with different disease duration and healthy controls. IAPP—amylin/islet amyloid polypeptide, proIAPP—proamylin/proislet amyloid polypeptide, CST—catestatin, ChgA—chromogranin A, NGF—nerve growth factor, PAF—platelet-activating factor, UMOD—uromodulin, I-FABP—intestinal fatty acid binding protein, yrs.—years, * *p*—probability value ≤ 0.05, ** *p*—probability value ≤ 0.001, and *** *p*—probability value ≤ 0.000001.

Except for UMOD and NGF levels, concentrations of tested substances were statistically significantly higher in the T1D group compared with the control group (Table 2). In the case of NFG, the differences between T1D and control groups were not significant, but the *p*-value was at the level of the statistical trend (*p* = 0.056). This trend was confirmed by a later analysis in more detailed subgroups (new cases vs. patients with long-term disease) (Figure 1).

When the levels of tested substances in children with newly diagnosed T1D were compared with the group of children with T1D treated for at least 3 years, it was shown that the duration of T1D affected the levels of NGF, ChgA, and PAF. 

The levels of PAF were significantly lower in newly diagnosed T1D compared with patients with T1D duration > 3 years: 0.20 (0.11–043) vs. 0.25 (0.12–5.18) ng/mL. In contrast, NGF and ChgA levels were significantly higher in newly diagnosed patients than in those treated over 3 years: 12.7 (3.45–17.9) vs. 4.69 (0.52–804) pg/mL for NGF and 74.5 (40.5–98.5) vs. 52.5 (15.5–104) ng/mL for ChgA, respectively).

### 3.3. The Effect of Disease Duration on the Selected Active Substances’ Concentrations in T1D Children

As we observed differences in T1D subgroups with shorter and longer disease duration, the whole T1D group was divided into three smaller ones (3–5 years, 6–7 years, and >7 years) and then reanalyzed. Figure 1 illustrates the differences between concentrations of active substances in these subgroups, the healthy control group, and the newly diagnosed patients. Detailed numerical data (median and range) and exact *p*-values are presented in the Supplementary Table S1. 

No effect of disease duration on IAPP, proIAPP, and I-FABP levels was observed, but concentrations of each substance statistically differed significantly between the T1D patients (in each subgroup) and the control group, except for UMOD. In contrast, concentrations of CST, ChgA, PAF, and NGF were statistically significantly influenced by the time that had passed since the diagnosis, but the direction of changes depended on the type of biomarker. 

In the case of ChgA—the highest concentrations were observed in patients just after the diagnosis of T1D (median 74.5 ng/mL, range 40.5–98.5) and they statistically differed significantly both from the control group (34.5 ng/mL *p* = 0.005) and the groups of patients treated for 3–5 years (52.5 ng/mL, *p* = 0.0001), 6–7 years (54.5 ng/mL, *p* = 0.0005, and >7 years (50.3 ng/mL, *p* = 0.000009). With the duration of the disease, ChgA concentration decreased, and in each time interval, it was statistically significantly lower compared to newly diagnosed patients. 

Although serum CST concentration did not differ between newly diagnosed T1D patients, with a median of 35.2 ng/mL (range 0.001–70.1), and those treated > 3 years, with a median of 20.1 ng/mL (range 0.001–305), the detailed analysis in subgroups of long-term patients showed the possible effect of disease duration. The highest concentrations of CST were observed in patients with newly diagnosed T1D; CST levels decreased in patients treated for 3–7 years, but 7 years after diagnosis, high values of the biomarker were observed again (Table S1). Statistically significant differences were observed between newly diagnosed patients and those treated for 3–5 years: *p* = 0.0008, median 35.2 ng/mL (range 0.000–70.1) vs. 19.7 ng/mL (0.007–95.6)*,* patients treated for 3–5 years and patients treated for >7 years: *p* = 0.0008, median 19.7 ng/mL (0.007–95.6) vs. 27.0 ng/mL (1.45–92.3), and the group treated for 6–7 years and those treated for >7 years: *p* = 0.019, median 20.0 ng/mL (0.005–305) ng/mL vs. 27.0 (1.45–92.3) ng/mL. 

In the case of PAF, patients with longer disease duration had higher concentrations of this substance (Figure 1, Table S1). A statistically significant difference (*p* < 0.05) was observed in the subgroups treated for 3–5 years (median 0.24 ng/mL, range 0.12–5.18), 6–7 years (median 0.25 ng/mL, range 0.18–2.67), and >7 years (median 0.29 ng/mL, range 0.12–3.9) in relation to those with newly diagnosed T1D (median 0.20 ng/mL, range 0.11–0.43).

The highest NGF concentration was observed in newly diagnosed patients: 12.7 pg/mL (3.45–17.9), followed by a decrease of values for patients treated for 3–5 years: 4.49 pg/mL (1.09–804), 6–7 years: 4.49 pg/mL (0.52–37.5), and after 7 years from diagnosis: 6.21 pg/mL (0.52–45.8). A statistically significant difference was observed only in patients newly diagnosed—in relation to the control group (*p* = 0.000004) and those who had been ill for 6–7 years (*p* = 0.002). 

## 4. Discussion

In a number of studies, selected biologically active substances were indicated as contributing to the etiology (IAPP [16,31,32,33,34,35,36], proIAPP [17,18,37], I-FABP [24]), course (CST [38,39], ChgA [40,41,42]) or the development of various complications (neuropathy—NGF [43,44,45], cardiovascular complications—PAF [26,46,47], and cardiovascular complications and nephropatia—UMOD [28,48,49]). In the current study, we assessed the diagnostic usefulness of these indicators present in T1D patients’ sera and confirmed statistically significant differences between their concentrations in the group of children with T1D and healthy children, but only some of them (NGF, ChgA, CST and PAF) were affected by disease duration. To the best of our knowledge, no one has analyzed concentrations of IAPP, proIAPP, CST, ChgA, NGF, PAF, UMOD, and I-FABP in the context of T1D duration. Our study showed that among the selected indicators, only UMOD did not show statistically significant differences between children with T1D and healthy children, but we found that UMOD levels in T1D patients were associated with serum creatinine concentration (r = −0.477, *p* < 0.005). Thus, this could suggest that UMOD should be rather assessed in urine than in the sera of T1D patients. 

Disease duration had no effect on IAPP and its precursor—proIAPP, as well as I-FABP, although the levels of these indicators were significantly higher in the sera of T1D patients compared to healthy children. An increased level of mature IAPP in the T1D group is opposite to results presented by Courtade et al. [17], but, like this researcher, we observed an elevated ratio of proIAPP to mature IAPP, which clearly indicates impaired proIAPP processing. It seems likely that IAPP aggregates, by inducing islet inflammation, may be a trigger or accelerator of autoimmunity in T1D. It is known that early prefibrillary aggregates that are difficult to observe histologically, may be present in the early stage of the disease, and the inflammatory properties of IAPP aggregates may play a role in the pathology of T1D [17,33,36,50]. 

Significantly elevated I-FABP levels in T1D patients, independent of disease duration, confirm our previous reports on epithelial damage in pediatric T1D and the utility of I-FABP as a serological marker of intestinal barrier dysfunction [24]. It is noteworthy that those substances whose concentration was not affected by the duration of the disease (IAPP, proIAPP, and I-FABP) are markers described as taking part in the pathomechanism of T1D.

Contrary to this observation, the concentrations of CST, ChgA, PST, and NGF varied in patients at different times since the onset of T1D. We did not observe one specific trend for all the indicated substances. This finding confirms previous reports showing that these substances could be associated with the T1D course and various late complications [26,38,39,40,41,42,43,44,45,46,47]. 

Our data on ChgA are consistent with the reports identifying ChgA as an autoantigen in T1D [19,51], and suggesting that altered ChgA levels may reflect changes in β—cell integrity [52]. Auto-reactive T cells targeting β-cell antigens are known to play a key role in β-cell destruction in T1D [53]. In this context, ChgA as an autoantigen localized in β—cell granules seems to be an attractive target for autoimmune reactions. Determination of ChgA concentration in patients’ sera may therefore potentially serve as an important biomarker of prediabetes. The highest concentration of ChgA observed by us in children with newly diagnosed T1D was also indicated by Xu et al. in adult T1D patients [52]. They reported that regular oral administration of verapamil in adult patients with T1D resulted in a decrease in ChgA levels, which remained at lower levels during treatment, and elevated levels of ChgA at the onset of the disease did not change in people from the control group who did not take verapamil. Our results in the group of healthy children are also consistent with their report, in which the level of ChgA in the serum of the healthy control group was about two times lower compared to those with T1D. Results opposite to our observations were obtained by Herold et al. [40], but it is worth noting that the researchers showed only a small but constant increase (*p* = 0.0410) in the level of ChgA depending on the duration of T1D. However, it should be noted that in this study, the duration of T1D in included patients was markedly longer (average 13.5 years) than in patients involved in our analysis. In addition, T1D patients with high levels of ChgA had enterochromaffin-like cell hyperplasia or autoimmune gastritis, i.e., conditions that were not found in the T1D children analyzed in our study. We speculate whether the lower concentrations observed in patients treated for more than 3 years are due to the breakdown of ChgA into protein cleavage products. The conditions under which ChgA decomposes are not fully known and described. Therefore, it would be worth assessing the concentration of other biologically active peptides, the precursor of which is ChgA: pancreastatin, WE-14, serpinin, and chromofungin [54,55]. Beta granule proteins (like ChgA) should not normally elicit an immune response, thus aberrant post-translational modification of the peptides is a possible hypothesis for how β—cell self-antigens are generated.

Unlike ChgA, there are no clear data on CST in T1D. It is known that CST is critical to maintaining metabolic and immune homeostasis by regulating immune cell infiltration and macrophage differentiation [56]. In addition, according to Ying et al., CST may be able to control hepatic glucose production, improve sensitivity to insulin, and have direct anti-inflammatory effects [57]. In our study, CST concentrations were statistically significantly higher in T1D patients than in the group of healthy people. Thus, this is in contrast to the observation of decreased CST levels in T2D patients previously reported by the researchers [57,58,59]. It should be emphasized, however, that the pathomechanism of both types of diabetes is different, and T1D should rather be compared with other autoimmune diseases. In this context, our results on CST concentrations during T1D are consistent with those shown in other autoimmunology diseases—inflammatory bowel disease [60]. In our study, the highest concentrations of CST were observed in patients with newly diagnosed T1D and those treated for more than 7 years. In patients who had been ill for more than 3 years but less than 7 years CST concentrations decreased. It would be desirable to investigate more closely whether the observed elevated levels in children with T1D are a compensatory mechanism for disturbances in glycemic homeostasis.

In the case of PAF, we showed that its concentrations were significantly higher in T1D patients who were ill for over 3 years compared to newly diagnosed patients, and this result is in line with other authors indicating that an increased level of PAF may indicate vascular complications in T1D patients. Cavallo-Perin et al. observed elevated PAF levels in patients with T1D and microalbuminuria, i.e., a manifestation of extensive vascular damage, and they suggested that PAF can be an indicator of micro- and macroangiopathy [46]. Nathan et al. indicate that elevated PAF levels may perpetuate hyperglycemia and promote or exacerbate micro- or macrovascular complications in T1D patients [26]. Ersoy et al. also emphasize that the high level of PAF detected in their study in T1D patients with long-term diabetes, compared to the group of healthy people, may be associated with vascular complications [47]. Since vascular complications are observed after many years of diabetes, and our pediatric patients did not present clinical vascular complications we suggest that PAF could be useful as a marker of early micro- and macrovascular changes in the course of T1D in children. However, further studies on T1D in children are needed to confirm this relationship. 

We also presented that the concentrations of NGF are influenced by T1D duration. The high NGF levels in newly diagnosed children can be explained by a stress-induced increase [61], and this result is in line with the observations of other researchers [62]. However, it should be emphasized that the observed decrease in NGF levels in the course of diabetes cannot be clearly interpreted. Many authors show that neurological complications induce a drop in serum NGF concentration [43,44,45]. It was shown that patients with T2D and neurological complications had lower levels of NGF compared to those without complications [63]. Moreover, there is no clear position on the NGF reference ranges for a healthy population. The concentrations described in the literature range from a few [64,65] to several dozen pg/mL [61,66,67]. Therefore, it is important to establish the norms of NGF for children and further research on this peptide.

## 5. Limitations and Strengths

To the best of our knowledge, this is the first study analyzing concentrations of IAPP, proIAPP, CST, ChgA, NGF, PAF, UMOD, and I-FABP in relation to diabetes duration in a pediatric population. 

However, we are aware that results should be interpreted with caution due to the relatively small amount of data on the concentrations of selected substances in the population of a healthy population. Due to the lack of these data, it is difficult to compare the obtained concentrations in the group of patients, especially since the literature reports are often contradictory. Evidently further studies are necessary in order to confirm the role of study indicators in the pathophysiology and course of T1D. 

We recognize that the presented results would be more accurate if we were able to observe patients over a longer period of time. In order to confirm our suggested conclusions, we plan to follow the patients participating in this study and perform the same tests again before our patients are 18 years of age. 

Exciting new questions and new answers may arise when we additionally analyze the tested substances in relation to glycemic control or biochemical parameters. 

## 6. Conclusions

The current study presented that concentrations of selected serum substances such as CST, ChgA, NGF, and I-FABP, prohormones/hormones (IAPP, proIAPP), and other active substances (PAF) differ between T1D patients and healthy controls. The level of some of them (CST, ChA, PAF, and NGF) have been shown to be dependent on the duration of T1D. However, further research is needed to confirm the role of these indicators in clinical practice, in particular in terms of using them as biomarkers in the course of diabetes in children and predicting long-term complications.

## Figures and Tables

**Table 1:** Biochemical status of T1D subgroups depending on the T1D duration.

Parameters	T1D (*n* = 156) ^a^	Newly Diagnosed (*n* = 30) ^b^	Disease Duration (in Years)		
			3–5 (*n* = 77) ^c^	6–7 (*n* = 25) ^d^	>7 (*n* = 24) ^e^
Blood glucose (mg/dL)	266 (75.6–792)	337 (153–792)	243 (119–640)	221 (144–569)	311 (75.6–512)
Glycated hemoglobin (%)	7.80 (5.90–18.8)	12.8 (6.60–18.8)	7.40 (6.10–14.3)	7.10 (5.90–10.5)	8.00 (5.9–11.5)
Serum peptide-C (ng/mL)	0.50 (0.06–3.78)	0.51 (0.30–3.22)	0.54 (0.20–3.78)	0.49 (0.19–2.15)	0.38 (0.06–0.88)
Serum insulin (mIU/L)	4.14 (2.00–21.9)	4.90 (3.39–21.9)	3.87 (2.00–11.9)	4.34 (3.00–7.34)	3.56 (2.00–6.89)
Serum total cholesterol (mg/dL)	158 (85.0–323)	156 (110–210)	159 (85.0–257)	168 (117–260)	159 (105–323)
Serum triglycerides (mg/dL)	70.0 (33.0–244)	86.0 (40.0–150)	61.0 (33–244)	77.0 (40–214)	73.0 (49.0–194)
Serum HDL cholesterol (mg/dL)	61.6 (23.5–115)	48.0 (23.5–99.5)	64.5 (26.2–98.5)	62.8 (40.4–90.2)	60.7 (47.7–115)
Serum LDL cholesterol (mg/dL)	82.5 (32.5–474)	88.5 (45.9–152)	82.2 (32.5–145)	90.0 (46.1–163)	76.3 (40.7–474)
Serum non-HDL cholesterol (mg/dL)	98.5 (41.9–267)	103 (57.9–168)	94.6 (41.9–186)	105 (55.1–194)	90.7 (50.5–267)
Serum CRP (mg/dL)	0.04 (0.03–0.53)	0.08 (0.03–0.48)	0.04 (0.03–0.53)	0.05 (0.03–0.15)	0.04 (0.03–0.11)
Serum witamin D (ng/mL)	27.9 (10.1–66.1)	29.5 (11.5–42.3)	29.5 (15.5–66.1)	25.3 (10.1–39.7)	26.2 (13.0–41.9)
Serum creatinine (mg/dL)	0.57 (0.23–1.05)	0.47 (0.23–0.85)	0.56 (0.33–1.01)	0.66 (0.38–1.05)	0.72 (0.47–1.05)

T1D—type 1 diabetes, n—sample size, HDL—high density lipoprotein, LDL—low density lipoprotein, CRP - c-reactive protein. Results are expressed as median and range (in brackets). Differences in the concentrations of the tested parameters between the groups significant at *p <* 0.05: blood glucose: ^a^ vs. ^b^, ^b^ vs. ^c^, and ^b^ vs. ^d^; glycated hemoglobin: ^a^ vs. ^b^, ^b^ vs. ^c^, ^b^ vs. ^d^, and ^b^ vs. ^e^; serum insulin: ^a^ vs. ^b^, ^b^ vs. ^c^, and ^b^ vs. ^e^; HDL: ^a^ vs. ^b^, ^b^ vs. ^c^, ^b^ vs. ^d^, and ^b^ vs. ^e^; serum triglycerides: ^a^ vs. ^b^ and ^b^ vs. ^c^; serum creatinine: ^a^ vs. ^b^, ^b^ vs. ^c^, ^b^ vs. ^d^, ^b^ vs. ^e^, and ^c^ vs. ^e^.

**Table 2:** Concentrations of selected active substances in T1D patients and healthy controls.

	T1D (*n* = 156)	T1D—Newly Diagnosed (*n* = 30)	T1D > 3 Years from Diagnosis (*n* = 126)	HC (*n* = 30)
IAPP [pg/mL]	75.0 (6.16–499) *p* < 0.000001	60.1 (17.3–148) *p* < 0.0000001	65.0 (6.16–499) *p* = 0.000005	33.5 (16.9–49.1)
		* p = 0.697		
proIAPP [pg/mL]	155 (34.8–958) *p* < 0.000001	99.6 (34.8–258) *p* < 0.0000001	92.6 (42.10–958) *p* = 0.000021	57.6 (19.5–82.3)
		* p = 0.513		
CST [ng/mL]	20.3 (0.001–305) *p* < 0.000001	35.2 (0.001–70.1) *p* = 0.003	20.1 (0.001–305) p = 0.617	20.2 (0.003–21.5)
		* p = 0.516		
ChgA [ng/mL]	55.7 (15.5–104) *p* < 0.000001	74.5 (40.5–98.5) *p* = 0.005	74.5 (40.5–98.5) *p* = 0.123	34.5 (11.5–88.0)
		* p < 0.000001		
NGF [pg/mL]	15.2 (0.52–804) *p* = 0.056	12.7 (3.45–17.9) *p* = 0.000004	4.69 (0.52–804) *p* = 0.036	4.30 (3.03–37.9)
		* p = 0.003		
PAF [ng/mL]	0.37 (0.11–5.18) *p* < 0.000001	0.20 (0.11–0.43) *p* = 0.588	0.25 (0.12–5.18) *p* = 0.194	0.19 (0.11–1.83)
		* p = 0.00001		
UMOD [ng/mL]	287 (39.0–875) *p* = 0.847	305 (90.5–610) *p* = 0.796	297 (39.0–875) *p* = 0.440	278 (51.0–555)
		* p = 0.601		
I-FABP [pg/mL]	970 (130–4560) *p* < 0.000001	1015 (280–2990) *p* = 0.000095	955 (130–4560) *p* = 0.000001	485 (170–2730)
		* p = 0.857		

T1D—type 1 diabetes, HC—healthy control, IAPP—amylin/islet amyloid polypeptide, proIAPP—proamylin/pro-islet amyloid polypeptide, CST—catestatin, ChgA—chromogranin A, NGF—nerve growth factor, PAF—platelet-activating factor, UMOD—uromodulin, I-FABP—intestinal fatty acid binding protein, *p* —probability value between T1D patients and healthy controls, * *p* —probability value between newly diagnosed T1D patients and T1D patients treated >3 years, and *p* < 0.05 was statistically significant; results are expressed as median and range (in brackets).

## Data Availability

The data presented in this study are available on request from the corresponding author. The data are not publicly available due to privacy protections.

## Supplementary Materials

The following supporting information can be downloaded at: https://www.mdpi.com/article/10.3390/jcm12062151/s1, Table S1: Selected markers in T1D patients with different disease duration and healthy controls—concentrations and significance of differences.
