# Supplementary material for: Selected Serum Markers Associated with Pathogenesis and Clinical Course of Type 1 Diabetes in Pediatric Patients—The Effect of Disease Duration
Source: J Clin Med. 2023 Mar 9;12(6):2151. doi: 10.3390/jcm12062151 (PMC10051659; doi:10.3390/jcm12062151)
Supplement: Supplementary file 1 [file jcm-12-02151-s001.zip › jcm-2191368-supplementary.pdf]

Table S1. Selected markers in T1D patients with different disease duration and healthy controls – concentrations and significance of differences.

IAPP [pg/ml]	newly diagnosed	3 - 5 yrs	6 - 7 yrs	> 7 yrs	control group
**newly diagnosed**	**60.1 (17.3 – 148)**				
**3 - 5 yrs**	*p = 0.419*	**53.8 (36.1 – 333)**			
**6 - 7 yrs**	*p = 0.499*	*p = 0.870*	**53.0 (25.1– 221)**		
**> 7 yrs**	*p = 0.226*	*p = 0.043*	*p = 0.077*	**80.9 (21.6 – 499)**	
**control group**	*p < 0.000001*	*p < 0.000001*	*p < 0.000001*	*p < 0.000001*	**33.5 (16.9 – 49.1)**
**proIAPP [pg/ml]**					
**newly diagnosed**	**99.6 (34.8 – 258)**				
**3 - 5 yrs**	*p = 0.204*	**90.5 (42.1 – 958)**			
**6 - 7 yrs**	*p = 0.306*	*p = 0.735*	**88.3 (55.7 – 452)**		
**> 7 yrs**	*p = 0.308*	*p = 0.092*	*p = 0.077*	**102 (58.9 – 352)**	
**control group**	*p < 0.000001*	*p < 0.000001*	*p < 0.000001*	*p <0.000001*	**57.6 (19.5 – 82.3)**
**CST [ng/ml]**					
**newly diagnosed**	**35.2 (0.001 – 70.1)**				
**3 - 5 yrs**	*p = 0.0008*	**19.7 (0.007 – 95.6)**			
**6 - 7 yrs**	*p = 0.384*	*p = 0.697*	**20.0 (0.005 – 305)**		
**> 7 yrs**	*p = 0.172*	*p = 0.0008*	*p = 0.019*	**27.0 (1.45 – 92.3)**	
**control group**	*p = 0.003*	*p = 0.707*	*p =0.565*	*p = 0.001*	**20.2 (0.003– 21.5)**
**ChgA [ng/ml]**					
**newly diagnosed**	**74.5 (40.5 – 98.5)**				
**3 - 5 yrs**	*p = 0.0001*	**52.5 (20.5 – 104)**			
**6 - 7 yrs**	*p = 0.0005*	*p = 0.572708*	**54.5 (22.5 – 94.5)**		
**> 7 yrs**	*p = 0.000009*	*p = 0.536305*	*p = 0.390*	**50.3 (15.5 – 98.0)**	
**control group**	*p = 0.005*	*p = 0.002*	*p = 0.054*	*p =0.001*	**34.5 (11.5 – 88.0)**
**NGF [pg/ml]**					
**newly diagnosed**	**12.7 (3.45 – 17.9)**				
**3 - 5 yrs**	*p= 0.079*	**4.49 (1.09 – 804)**			
**6 - 7 yrs**	*p = 0.002*	*p = 0.508*	**4.49 (0.52 – 37.5)**		
**> 7 yrs**	*p= 0.492*	*p = 0.164*	*p =0.153*	**6.21 (0.52 – 45.8)**	

**control group**	$p= 0.000004$	$p= 0.553$	$p = 0.735$	$p = 0.089$	**4.30 (3.03 – 37.9)**
**PAF [ng/ml]**					
**newly diagnosed**	**0.20 (0.11 – 0.43)**				
**3 - 5 yrs**	$p = 0.000633$	**0.24 (0.12 – 5.18)**			
**6 - 7 yrs**	$p = 0.000995$	$p = 0.284$	**0.25 (0.18 – 2.67)**		
**> 7 yrs**	$p = 0.002042$	$p = 0.119$	$p = 0.435$	**0.29 (0.12 – 3.9)**	
**control group**	$p = 0.588$	$p = 0.000003$	$p = 0.000005$	$p = 0.000113$	**0.19 (0.11 – 1.83)**
**UMOD [ng/ml]**					
**newly diagnosed**	**305 (90.5 – 610)**				
**3 - 5 yrs**	$p = 0.969$	**297 (39.0 – 875)**			
**6 - 7 yrs**	$p = 0.565$	$p = 0.560$	**275 (80.5 – 465)**		
**> 7 yrs**	$p = 0.347$	$p = 0.340$	$p = 0.603$	**254 (104 – 673)**	
**control group**	$p = 0.796$	$p = 0.895$	$p = 0.600$	$p = 0.309$	**278 (51.0 – 555)**
**I-FABP [pg/ml]**					
**newly diagnosed**	**1015 (280 – 2990)**				
**3 - 5 yrs**	$p = 0.898$	**960 (260 – 4560)**			
**6 - 7 yrs**	$p = 0.220$	$p = 0.118$	**790 (130 – 2900)**		
**> 7 yrs**	$p = 0.365$	$p = 0.317$	$p = 0.061$	**1140 (440 – 3450)**	
**control group**	$p = 0.0001$	$p < 0.000001$	$p = 0.003$	$p = 0.000008$	**485 (170 – 2730)**
